# Inner myometrial laceration complicated by severe antepartum haemorrhage: a narrative review and case report

**DOI:** 10.1186/s12884-025-08382-6

**Published:** 2025-11-10

**Authors:** Yin Wang, Dehong Liu, Xiumei Wu, Xianxia Chen

**Affiliations:** Department of Obstetrics and Gynecology, Maternal and Child Medical Center of Anhui Medical University, Hefei, Anhui 230001 China

**Keywords:** Inner myometrial laceration, Antepartum haemorrhage, Refractory haemorrhage, Caesarean section, Intraoperative diagnosis, Primary suture

## Abstract

**Background:**

Inner myometrial laceration (IML) is a rare but potentially life-threatening obstetric emergency that can cause severe antepartum or postpartum haemorrhage. The pathogenesis of this condition is not fully understood, and this condition is often associated with abnormal uterine contractions, fetal position factors, or obstetric interventions. Its clinical manifestations are nonspecific, making early diagnosis difficult and leading to potential misdiagnosis or missed diagnosis.

**Case presentation:**

This report describes a 26-year-old primigravida at 38⁺¹ weeks gestation who underwent oxytocin induction for preeclampsia. During induction, she suddenly developed 1200 ml of vaginal bleeding. An emergency caesarean section revealed a 4 cm inner myometrial laceration on the posterior wall of the lower uterine segment, with the serosal layer intact. Haemostasis was successfully achieved using a “figure-of-8” suture combined with a continuous suture, supplemented with bilateral ligation of the ascending branches of the uterine arteries. The patient recovered well postoperatively, with no complications during follow-up, and her uterus was preserved.

**Conclusion:**

IML is an important and occult cause of refractory antepartum or postpartum haemorrhage. Diagnosis relies on careful intraoperative exploration. Individualized suturing techniques and necessary vascular ligation are key to preserving fertility, whereas hysterectomy should be reserved as a last resort when conservative measures fail. Enhancing clinical vigilance for IML, early surgical exploration, and targeted repair is crucial for improving maternal and fetal outcomes.

## Introduction

Postpartum haemorrhage (PPH) is among the leading causes of maternal mortality worldwide, and antepartum haemorrhage (APH) also seriously threatens maternal and fetal safety [1]. In addition to maternal coagulation disorders, vaginal and cervical lacerations, uterine rupture, placental abruption, and placenta previa [2], inner myometrial laceration (IML)—a rare and occult cause of both antepartum and postpartum haemorrhage—is gradually gaining clinical attention [3]. IML specifically refers to pathological injury in which the endometrium and part of the myometrium are torn but the serosal layer remains intact. Its occurrence is often related to factors such as excessive uterine contractions, abnormal fetal position, or the use of oxytocic drugs [4]. Since Hayashi et al. [5] first systematically described it in 2000, only sporadic case reports have been published globally. Owing to the lack of specific clinical manifestations, preoperative diagnosis is extremely difficult; most cases are diagnosed during a caesarean section or via pathological examination after hysterectomy performed for refractory haemorrhage [4]. Therefore, improving awareness of IML, early recognition, and timely surgical intervention are highly important for reducing hysterectomies and preserving patient fertility. This article reports a case of antepartum haemorrhage caused by IML and presents a review of the literature to explore the clinical features and diagnostic aspects of this condition, as well as management strategies, providing reference information for clinical prevention and treatment.

## Case presentation

The patient was a 26-year-old primigravida at 38 + 1 weeks gestation, admitted to the hospital for planned delivery because of preeclampsia. She had no history of uterine surgery. She had 11 regular prenatal check-ups during pregnancy. A routine prenatal check-up at 37 + 6 weeks of gestation revealed a blood pressure of 138/95 mmHg, along with a 24-hour urinary protein excretion of 460 mg, raising the suspicion of preeclampsia. She was subsequently admitted to our hospital at 38 + 1 weeks for planned delivery. Upon admission, her blood pressure was recorded at 138/100 mmHg, a heart rate of 89 beats/min, and a temperature of 36.5 °C. Laboratory tests revealed a haemoglobin concentration of 123 g/L, a platelet count of 179 × 10⁹/L, coagulation function (PT of 11.1 s, APTT of 26.6 s, and fibrinogen concentration of 4.14 g/L) and liver and kidney function (ALT concentration of 20 U/L and Cr concentration of 40.9 µmol/L) within the normal range.

**Fig. 1:**
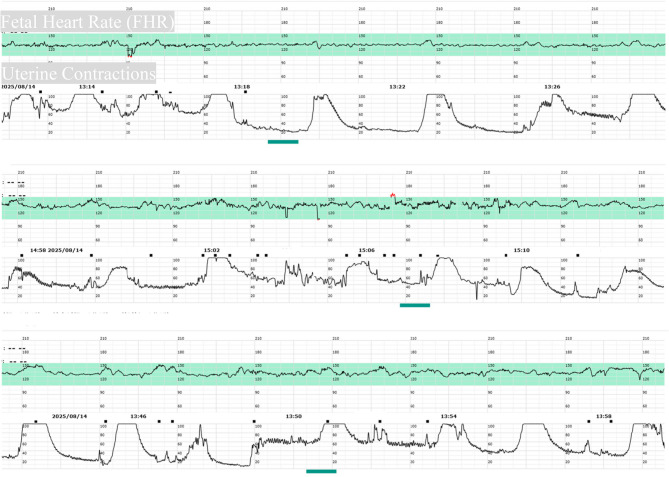
Continuous cardiotocography (CTG) tracing during oxytocin infusion for labor induction

The cervical Bishop score on admission was 6 (evaluating cervical dilation, effacement, consistency, position, and fetal station). Intravenous oxytocin induction was initiated, which failed on the first day. Intravenous oxytocin induction was initiated according to our institutional protocol (detailed in Table 1. On the second day, she was sent to the delivery room again for intravenous oxytocin induction. At 10:15, a 1% oxytocin infusion was started with continuous fetal heart monitoring (Fig. 1). At 15:56, the patient complained of vaginal fluid leakage. After routine disinfection, a vaginal examination was performed. The cervix was soft but not dilated, with a cervical canal length of 0.5 cm. The membranes were intact, and the presenting part was the vertex at station − 2. The instantaneous vaginal blood loss was approximately 1200 ml and was bright red. Oxytocin was immediately stopped. An emergency caesarean section under general anaesthesia was performed in the delivery room operating theatre.

**Fig. 2:**
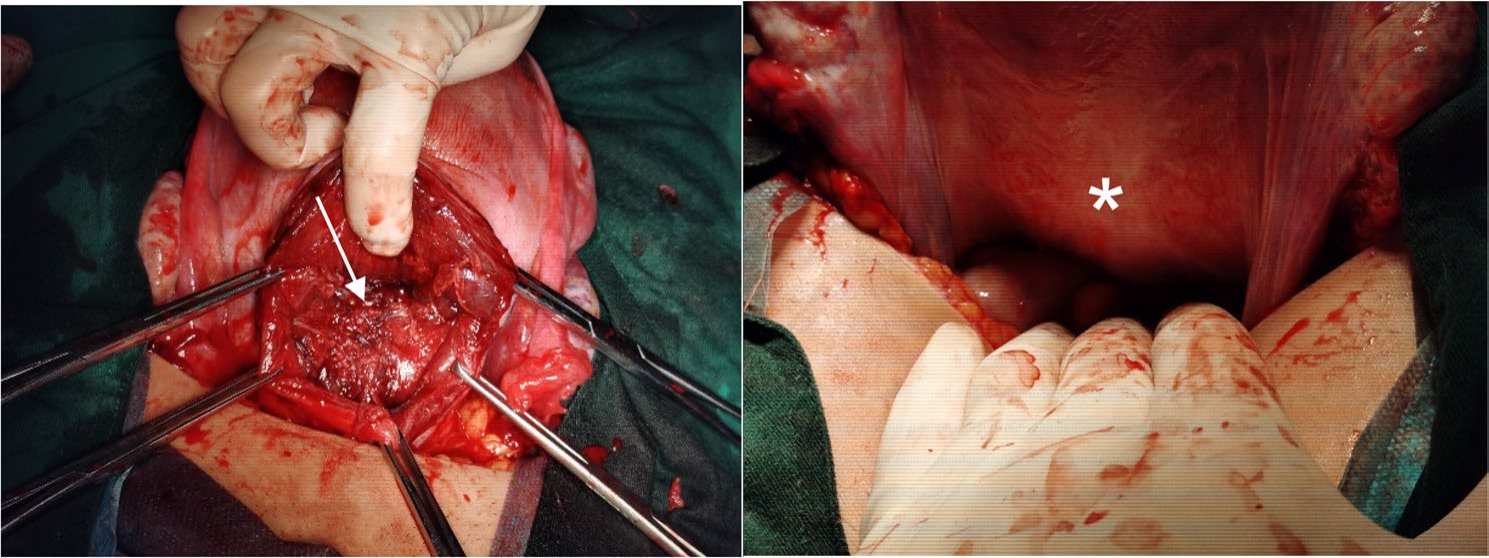
Intraoperative photograph of the inner myometrial laceration. The view is through the hysterotomy incision during cesarean section, looking toward the posterior wall of the lower uterine segment. The arrows delineate the margins of a longitudinal laceration, approximately 4 cm in length. The depth of the laceration extends through approximately two-thirds of the myometrial thickness. Critically, the overlying uterine serosa is intact and appears normal (asterisk, *). This finding confirms the diagnosis of an inner myometrial laceration rather than a uterine rupture

A transverse lower abdominal incision was made. The uterus was opened via a transverse incision in the lower uterine segment. Clear amniotic fluid, approximately 500ml, was noted. The placenta was located on the fundal posterior wall, with no signs of abruption or accreta. Twenty units of oxytocin were injected into the uterine body. The placenta separated completely and spontaneously after 1 min. At this time, the uterine body contracted well, but copious amounts of bright red blood gushed from the lower uterine segment. After the lower uterine segment was packed with large gauze pads and the abdominal incision was covered with wet gauze, the patient was placed in the lithotomy position. Exploration with a vaginal speculum revealed no active bleeding; examination revealed no lacerations in the vagina or cervix. Upon re-exploration in the abdomen, there was no haemoperitoneum, and the uterine body was well contracted. When the large gauze pressure pad covering the lower uterine segment was removed, bright red blood immediately spurted out. The uterus was delivered out of the pelvic cavity. Careful examination revealed a longitudinal inner myometrial laceration at the 6 o’clock position on the posterior wall of the lower uterine segment, approximately 4 cm long, extending 2/3 through the myometrial thickness, with the serosal layer intact (Fig. 2).

**Fig. 3:**
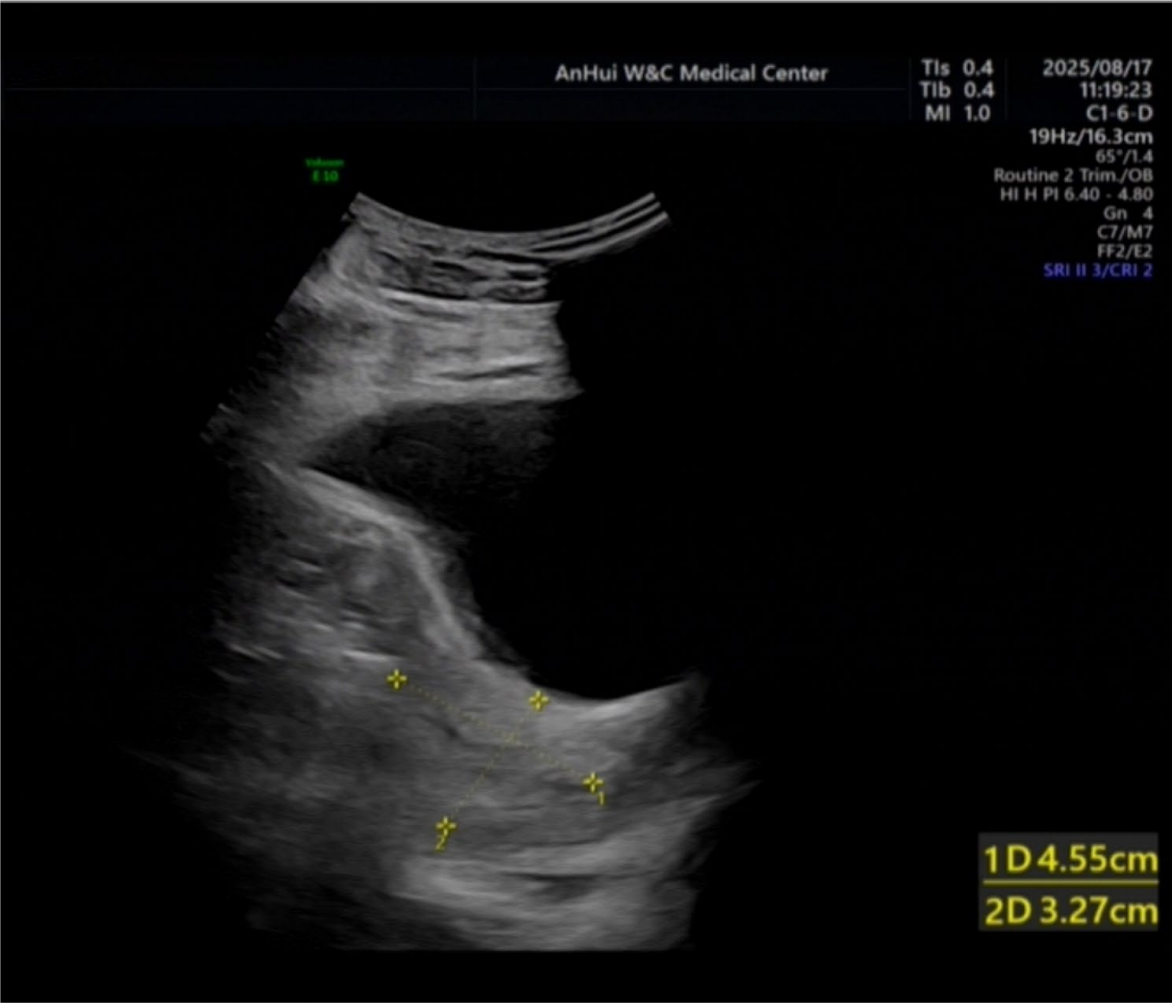
Postoperative transvaginal ultrasound image obtained one week after surgery.This sagittal view shows the lower uterine segment at the site of the repaired laceration and hysterotomy. The arrowheads point to the region of the healed scar, demonstrating a continuous and intact myometrial layer with no evidence of defect or hematoma. The endometrial stripe is visible and appears normal. These findings indicate satisfactory early healing following primary repair

A 1–0 absorbable suture (Vicryl) was used to first place a “figure-of-8” suture at the base of the laceration, followed by a continuous suture to reinforce the muscle layer. Bilateral ligation of the ascending branches of the uterine arteries was also performed simultaneously, without uterine packing. The total estimated blood loss was 1600 ml, comprising approximately 1200 ml of antepartum vaginal blood loss and 400 ml of intraoperative loss (estimated by weighing surgical sponges and measuring suction volume). Postoperative monitoring revealed that the haemoglobin concentration decreased to 77 g/L. Given that the patient was haemodynamically stable with no signs of continued bleeding, transfusion was not administered. The patient recovered well and was discharged 72 h post-operatively. One-week follow-up revealed that the patient had no complaints of abnormal vaginal bleeding or other discomfort. Ultrasound revealed a well-healed uterine scar, a continuous and intact myometrium, and no abnormal blood flow signals (Fig. 3). As shown in Table 1.

**Table 1:** Summary of clinical timeline and management

Timeline (Gestational Age)	Clinical Event/Intervention	Key Findings/Vital Signs
37 + 6 weeks	Routine prenatal check-up	BP: 138/95 mmHg; 24-h urine protein: 460 mg
Admission (38 + 1 weeks)	Admission for preeclampsia	BP: 138/100 mmHg; HR: 89 bpm; Hb: 123 g/L; Bishop Score: 6
Day 1 of Admission	First attempt of oxytocin induction	Failed induction
Day 2 of Admission (~ 10:15)	Start of second oxytocin induction	Regimen: [2.5 IU of oxytocin in 500 mL of Lactated Ringer’s solution, starting at 2 mIU/min and increasing by 2 mIU/min every 30 min.]
Day 2 (~ 15:56)	Sudden massive vaginal hemorrhage	Estimated blood loss: 1200 ml; Membranes intact; Cervix not dilated
Following minutes	Emergency management & transfer	Oxytocin stopped; Emergency cesarean section decided
Intraoperative	Cesarean section & discovery of IML	4 cm laceration on posterior lower uterine segment; Serosa intact
Postoperative (72 h)	Hospital discharge	Patient stable, Hb: 77 g/L
1-week follow-up	Outpatient follow-up	Well-healed uterine scar on ultrasound; patient asymptomatic

## Discussion

In 2000, Hayashi et al. [5] reported IML in three patients with postpartum haemorrhage that was refractory to conventional uterine atony treatment. Based on observations of hysterectomy specimens, a hypothetical uterine corpus model, and data from 34 women, they proposed IML as a new cause of postpartum haemorrhage, suggesting that it results from excessive stress on the cervix due to abnormally high intrauterine pressure during delivery. In 2016, Kaplanoglu et al. [4] first explicitly listed IML as a cause of antepartum haemorrhage based on four patients who bled during the active phase of labour and were diagnosed with IML via uterine incision during caesarean section after other causes of bleeding were excluded. In 2024, Khakifirooz et al. [6] reported a rare case of massive intrapartum haemorrhage in which the patient survived because of timely diagnosis and management of a myometrial laceration. This patient was a 26-year-old primigravida admitted at term with premature rupture of membranes and vaginal bleeding. During spontaneous labour without oxytocin, the estimated blood loss reached 750 ml at 5 cm of cervical dilation. An emergency caesarean section revealed a 4 cm longitudinal laceration in the inner layer of the myometrium on the posterior wall of the lower uterine segment. Haemostasis was achieved with a continuous locked absorbable suture, and the patient recovered well.

The case discussed in the present report represents the third report related to IML causing antepartum haemorrhage: a patient at 37 weeks gestation receiving oxytocin induction for preeclampsia who developed sudden antepartum haemorrhage (estimated blood loss of 1200 ml) before cervical dilation, leading to an emergency caesarean section. Intraoperatively, a 4 cm laceration was found within the myometrium of the posterior lower uterine segment (serosa intact), and haemostasis was successfully achieved using absorbable sutures in a “figure-of-8” plus continuous fashion. The patient recovered well postoperatively.

A pertinent question is whether IML constitutes a distinct entity or merely represents uterine dehiscence described with different terminology. Uterine dehiscence typically denotes a myometrial defect that extends through the majority of the uterine wall thickness, leaving only the serosal layer intact; it is a precursor to complete rupture and is often associated with a scarred uterus or obstructed labor [7]. In contrast, the anatomical distinction of IML underlies a different clinical presentation and implication: the hallmark of IML is sudden, torrential hemorrhage from the denuded inner vasculature, often with a well-contracted uterine body and less dramatic pain. Uterine dehiscence, however, more frequently presents with signs of impending catastrophe, such as severe pain, fetal heart rate abnormalities, and hemodynamic instability due to the profound weakening of the uterine wall [7, 8]. Therefore, IML is not a semantic play but a distinct lesion with a specific anatomical substrate, pathophysiology (shear stress in the inner wall versus full-thickness wall failure), and clinical implications. Recognizing IML as a separate entity is crucial, as it mandates a specific search pattern during intraoperative exploration for refractory hemorrhage—focusing on the endometrial surface of the lower uterine segment—to avoid missed diagnosis.

The incidence of IML remains unclear. Since the diagnosis can be confirmed only by direct examination of hysterectomy specimens or via a uterine incision, cases may be missed. Additionally, some minor lacerations might have been successfully managed with uterine packing [9].

The apparent rarity of IML in published reports may reflect not only its true biologic infrequency but also a degree of global underrecognition and misclassification. Prior to the description by Hayashi et al. [5], many cases of sudden hemorrhage with intact serosa may have been coded as incomplete rupture or uterine dehiscence. Even in more recent literature, diagnostic overlap persists because intraoperative exploration is not always meticulous enough to identify a superficial laceration confined to the inner myometrium and many obstetricians are entirely unaware of the disease entity. This underreporting bias likely explains why so few cases are available in the literature despite the plausibility of higher clinical occurrence.

The pathogenesis of inner myometrial laceration (IML) has not been fully elucidated. Among previously reported cases with clearly documented laceration sites, approximately 80% occurred on the posterior uterine wall [9]. This predilection for the posterior wall and lateral walls of the lower uterine segment suggests that the abnormal distribution of intrauterine pressure might be one injury mechanism [5]. Furthermore, most cases are associated with the use of oxytocic drugs or augmented contractions, indicating that the occurrence of IML may be linked to abnormal contraction patterns or obstetric interventions [9]. The case reported in this article involves a patient admitted for preeclampsia who underwent oxytocin-induced labor. Before the occurrence of antepartum hemorrhage, the patient had exhibited significant signs of uterine hyperstimulation.

The primary sign of IML is unexplained massive antepartum or postpartum haemorrhage. The patient may have received potent uterotonics (e.g., oxytocin, carboprost), and although the uterus may feel well contracted on external examination, bright red blood continues to flow heavily from the vagina and responds poorly to medication [3–9, 11, 12]. Pain may be present but is typically less severe and less dramatic than in complete uterine rupture.

Diagnosing IML involves many challenges. Sudden massive haemorrhage is its hallmark, often manifesting as unprovoked vaginal bleeding during active labour or after placental delivery, with blood loss rapidly reaching over 1000 ml and a poor response to conventional uterotonics (e.g., oxytocin and carboprost) [9]. Definitive diagnosis relies primarily on intraoperative exploration. After excluding common causes, caesarean section or laparotomy is the gold standard for diagnosing IML. Typical intraoperative findings include: (1) an intact serosal layer, but with a longitudinal laceration in the myometrium, extending up to 2/3 of its depth; and (2) lacerations more commonly located on the posterior and lateral walls, mostly single, though cases with multiple or unspecified sites have been reported [4]. Although pathological examination can reveal characteristic changes such as muscle fibre breaks with haemorrhage, it is seldom used in emergency settings [5].

In terms of differential diagnosis, IML must be distinguished from (1) placental abruption, which typically presents with abdominal pain, a rigid abdomen, and ultrasound showing a retroplacental haematoma [13]; (2) uterine rupture, which presents as a full-thickness tear with intra-abdominal bleeding and often leads to shock [8]; and (3) cervical laceration, where the cervical wound can be directly observed via vaginal examination [14].

The management strategy for IML depends on the number and location of lacerations and the severity of bleeding. Commonly reported treatments in the literature include hysterectomy, primary suture, and various combined procedures [9]. Hysterectomy is often used for multiple or severe lacerations combined with uncontrollable bleeding [4, 5, 9, 12]; primary suture is commonly used for localized single lacerations [4, 6, 9, 12], with some cases combined with uterine artery ligation [4], B-Lynch suture [4, 9], Cho compression suture [3], or internal iliac artery ligation [11]to enhance haemostasis.

With respect to primary suturing techniques, it is recommended to choose the appropriate method based on the injury characteristics: (1) layered suture should be the first choice, using 2–0 or 3–0 absorbable suture (e.g., Vicryl), first placing a “figure-of-8” suture to close the base of the laceration, followed by continuous suturing to reinforce the muscle layer to avoid dead space and reduce rebleeding risk [10]; (2) if the laceration involves branches of the uterine artery, ligation of the ascending branch of the uterine artery (BUtAL) should be combined to effectively control bleeding [9]; and (3) for diffuse myometrial injury, compressive suturing techniques such as B-Lynch suture [15] or Cho square suture can be considered to compress the uterine volume and reduce the blood supply [3]. (4) All procedures should be performed with the patient in the lithotomy position to allow real-time observation of vaginal bleeding [3, 4].

In previous cases where hysterectomy was performed for IML, the diagnosis was often confirmed only by postoperative pathological examination following uncontrollable massive postpartum haemorrhage or haemorrhagic shock. If the possibility of IML is considered early and timely surgical exploration with primary suturing is performed, most patients can achieve successful haemostasis through meticulous suturing, thereby avoiding hysterectomy. The indications for hysterectomy should be strictly limited to the following situations: persistent active bleeding after suturing that threatens the patient’s life, or concomitant irreparable uterine rupture and placenta accreta [3].

## Conclusion

Inner myometrial laceration (IML) is a rare but important cause of life-threatening antepartum or postpartum haemorrhage. Its occurrence is closely related to abnormal uterine contractions, fetal head pressure, or iatrogenic intervention, particularly in the posterior wall of the lower uterine segment. IML typically presents as sudden, refractory vaginal haemorrhage that responds poorly to conventional uterotonics, with diagnosis requiring intraoperative exploration. Treatment should be individualized based on the extent of the laceration and severity of bleeding. Primary meticulous suturing combined with necessary vascular ligation is key to preserving fertility, whereas hysterectomy should be strictly reserved for failure of conservative treatment or when there is associated irreparable damage. Enhancing the clinical recognition of IML and awareness of intraoperative exploration is recommended to improve prognosis and reduce the risk of hysterectomy.

## Data Availability

Not applicable.
